# Projected Health and Economic Benefits of Air Quality Targets in China: Modeling Study

**DOI:** 10.2196/84809

**Published:** 2026-04-01

**Authors:** Siyuan Wang, Zhiwei Xu, Gian Luca Di Tanna, Yawen Jiang, Mingsheng Chen, Laura Downey, Stephen Jan, Lei Si

**Affiliations:** 1 The George Institute for Global Health, UNSW, Sydney, New South Wales, Australia; 2 School of Medicine and Dentistry, Griffith University, Gold Coast, Queensland, Australia; 3 Department of Business Economics, Health and Social Care, University of Applied Sciences and Arts of Southern Switzerland, Lugano, Switzerland; 4 School of Public Health (Shenzhen), Sun Yat-Sen University, Shenzhen, China; 5 School of Health Policy and Management, Nanjing Medical University, No. 101 Longmian Avenue, Nanjing, 211166, China, 86 02586868506; 6 Jiangsu Health Vocational College, Nanjing, China; 7 Faculty of Medicine, Imperial College, London, United Kingdom; 8 School of Health Sciences, Faculty of Health, Western Sydney University, Campbelltown, New South Wales, Australia; 9 School of Health Management, Anhui Medical University, Hefei, China

**Keywords:** China, health impact modeling, particulate matter, economic benefits, air pollution

## Abstract

**Background:**

Air pollution continues to impose a substantial health and economic burden in China. Despite recent improvements, national annual average PM2.5 (fine particulate matter) concentrations remain substantially above the levels deemed safe by the World Health Organization (WHO), underscoring the need for more stringent air quality control.

**Objective:**

This study aims to quantify the projected health and economic benefits of reducing PM2.5 concentrations in China under the Healthy China 2030 plan.

**Methods:**

Using the 2020 ground-level PM2.5 data as a baseline, we projected cause-specific mortality and morbidity outcomes for 337 prefecture-level cities in China from 2020 to 2030 under four policy scenarios: (1) Healthy China 2030 (10% reduction by 2025; 25 µg/m³ by 2030) and the WHO targets of (2) 15 µg/m³, (3) 10 µg/m³, and (4) 5 µg/m³ by 2030. Mortality for noncommunicable diseases, lower respiratory infections, stroke, ischemic heart disease, lung cancer, and chronic obstructive pulmonary disease was estimated using the Global Exposure Mortality Model. Hospitalizations were modeled using log-linear models based on national evidence.

**Results:**

In 2020, PM2.5 concentrations across the 337 cities ranged from 7 to 63 µg/m³, with a national annual mean of 32.6 µg/m³. Higher concentration levels were observed in eastern China, particularly in the eastern and southeastern regions. Overall, our analysis accounted for nearly 70% of the total population of China in 2020. Our analysis shows that maintaining PM2.5 at 2020 levels was projected to result in 9.04 million (95% CI 7.70‐10.67 million) attributable deaths, compared with 8.63 million (95% CI 7.42‐10.30 million) under the policy scenario, corresponding to 0.41 million premature deaths averted under the 14th Five-Year Clean Air Plan. Over 2021‐2030, PM2.5-attributable deaths declined from 17.76 million (95% CI 14.21‐20.95 million) under baseline conditions to 15.96 million (95% CI 12.85‐19.15 million) under the policy scenario. Achieving WHO targets would further reduce attributable deaths to 13.99 million (95% CI 11.25‐15.25 million) at 15 µg/m³, 12.86 million (95% CI 10.85‐14.85 million) at 10 µg/m³, and 11.49 million (95% CI 8.96‐13.45 million) at 5 µg/m³. The annual average hospitalizations declined by 21,422 cardiovascular and 26,545 respiratory admissions under the policy scenario, increasing to 41,690 and 51,681 at 15 µg/m³, 51,884 and 64,333 at 10 µg/m³, and 62,146 and 77,073 at 5 µg/m³, respectively. Subsequently, total economic gains reached US $123.7 billion under the policy scenario and increased to US $185.7 billion, US $240.7 billion, and US $306.5 billion under the 15, 10, and 5 µg/m³ scenarios, respectively.

**Conclusions:**

Our findings suggest that while the Healthy China 2030 Plan offered substantial health gains, achieving stricter WHO air quality targets could yield 2-3 times greater benefits. These findings will support the future development of stricter evidence-based national air quality standards.

## Introduction

Ambient air pollution represents a major global environmental health challenge. In 2019, the World Health Organization (WHO) estimated that exposure to outdoor air pollution was responsible for more than 4.2 million premature deaths worldwide [1]. In particular, there is a growing body of epidemiological evidence linking exposure to fine particulate matter (PM) with increased risks of cardiovascular and respiratory diseases, lung cancer, and premature mortality [2-6]. In addition to the substantial health burden, exposure to PM2.5 also imposes considerable economic costs, including direct health care expenditures from air pollution–induced illnesses, increased hospitalizations, and indirect losses from reduced workforce productivity [7]. In particular, the burden falls disproportionately on low- and middle-income countries, where around 80% of individuals exposed to unsafe annual PM2.5 concentrations reside and nearly 90% of air pollution–related premature deaths occur [1,8]. Recognizing the increasing health and economic burden associated with exposure to elevated levels of PM, many low- and middle-income countries have introduced national policies aimed at reducing PM levels. For example, countries in Southeast Asia, such as Indonesia, Vietnam, and Thailand, have implemented mitigation strategies to varying degrees, including the establishment of ambient air quality standards, the introduction of emission controls across key sectors, such as transport, industry, and energy, and the strengthening of air quality monitoring systems, with the aim of reducing both emission levels and population exposure [9-11]. In India, the National Clean Air Program has been associated with substantial improvements in urban air quality, with declines in PM10 concentrations across 20 major cities, and is estimated to have averted approximately 62,219 excess mortalities in 2021 compared to 2018 [12].

In China, ambient air pollution remains a critical public health issue, contributing to an estimated 1.67 million premature deaths nationwide in 2020 [13]. Exposure to PM is the nation’s fourth leading risk factor for overall mortality and disability-adjusted life years [14]. Overall, this contributes substantially to both the health and economic burden, with several studies estimating associated annual economic losses ranging from 0.92% to 3.6% of the national gross domestic product (GDP) within the past decade [13,15,16]. The Chinese government introduced the Air Pollution Prevention and Control Action Plan in 2013, setting ambitious targets to reduce pollution levels [17]. The plan aimed to lower PM concentrations in prefecture-level cities and above by at least 10% from 2012 levels by 2017. Consequently, the annual average PM2.5 concentration in 74 major cities declined by 33.3%, from 72.2 μg/m³ in 2013 to 47.0 μg/m³ in 2017 [18]. This improvement has resulted in substantial health benefits, including the reduction of 47,240 deaths and the prevention of 710,020 years of life lost [18].

In 2021, the WHO updated its air quality guidelines, lowering the recommended annual average PM2.5 concentration to 5 μg/m³ [19]. This revision reflects a growing body of epidemiological evidence demonstrating that both short-term and long-term exposure at low threshold levels to PM can still result in adverse health effects [3,4,20]. Despite significant improvements in air quality, PM2.5 levels in 2020 remained 7 times higher than the updated recommended limit. Han et al [21] estimated that long-term exposure to ambient PM2.5 above the new WHO guideline was associated with approximately 1.4 million premature deaths and US $1006.9 billion in the total value of statistical life annually. As part of its commitment to building a "Beautiful China," the government introduced a new set of air quality standards under the latest Clean Air Action Plan, aiming to reduce the annual mean PM2.5 concentration by 10% between 2020 and 2025 and achieving the interim target of 25 µg/m³ by 2030 [22,23]. This initiative underscores China’s ongoing efforts to mitigate the health and economic burdens associated with air pollution.

Currently, much is known about the national health and economic disease burden associated with PM2.5 exposure. For example, Maji et al [13] estimated the disease burden associated with PM2.5 across 338 cities, reporting that PM2.5-attributable mortality reached 0.964 million in 2016, accounting for 9.98% of total reported deaths in China that year. Other national-level estimates since 2010 have ranged from 1.02 to 2.20 million deaths [24-28]. Additionally, several studies have also retrospectively examined the benefits of previous air quality guidelines or policies, either nationally or at a regional level. Xu et al [29] assessed the health and economic impacts of clean air policies in the Beijing-Tianjin-Hebei region, estimating a reduction of 24,000 premature deaths and associated economic benefits of US $60 billion. Zhang et al [30] compared the benefits of the previous 5-year Clean Air Action Plan with its implementation costs, reporting that the public health benefits of air quality improvements were 1.5 times greater than the mitigation costs. However, the understanding of the potential benefits of future air quality improvements, particularly those projected under the latest clean air policies, remains limited. Given the significant gap between current national air quality standards and WHO recommendations, evaluating the health and economic impacts of national air policies is essential for supporting evidence-based decision-making as China advances toward its Healthy China 2030 goals. To bridge this dearth of evidence, we modeled the attributable health and economic outcomes associated with achieving the latest national regulations from 2021 to 2030, as well as the additional benefits associated with reaching the more stringent WHO targets of 15, 10, and 5 µg/m³ over the same period from a health system perspective.

## Methods

### Policy Scenarios and Model Period

We projected cause-specific mortality and morbidity outcomes for 337 prefecture-level cities in China from 2020 to 2030 under several air quality improvement scenarios. These include (1) a 10% reduction in PM2.5 by 2025, targeting 25 µg/m³ by 2030, representing the policy scenario under the current clean air action plan; (2-3) a stepwise reduction to WHO interim targets, with annual PM2.5 concentration levels reaching 15 µg/m³ (Interim Target III), and 10 µg/m³ (Interim Target IV) by 2030, respectively; and (4) achieving the WHO’s recommended air quality guideline of 5 µg/m³ by 2030. To account for population changes, age-specific and sex-specific baseline population data were projected for the modeled period under shared socioeconomic pathways for each of the air quality scenarios. Table S1 in Multimedia Appendix 1 presents the matrix summarizing the air quality scenarios and population projections considered in this analysis.

### Statistical Analysis

We used the widely used health impact function to model mortality and morbidity outcomes under the policy scenarios [31]. Specifically, the health impact function is given as follows:


$$\Delta y=x_{0}\times \frac{\left(RR-1\right)}{RR}\times Pop$$

where the health end point attributable to PM2.5 ∆y) is a function of the baseline incidence rate $x_{0}$, the population attributable risk [(RR-1)/RR], and the population at risk (Pop). Mortality outcomes included the combined category of noncommunicable diseases and lower respiratory infections (NCD + LRI), alongside 5 specific causes: stroke, ischemic heart disease (IHD), chronic obstructive pulmonary disease (COPD), lung cancer (LC), and LRIs. Morbidity outcomes assessed included hospital admissions related to cardiovascular and respiratory conditions.

### Mortality Attributable to PM2.5 Exposure

Mortality attributable to PM2.5 exposure under baseline and policy scenarios was estimated using the global exposure mortality model (GEMM), which provides age-specific and cause-specific hazard ratios for nonaccidental mortality [32]. The GEMM is a multivariate random effects model that synthesizes evidence from 41 cohort studies globally to characterize the exposure-response relationship between ambient air pollution and nonaccidental mortality and has been widely applied in health assessment studies of ambient air pollution internationally. Details of the model have been described elsewhere [32]. In brief, the GEMM characterizes the exposure-response relationship as:


$$GEMM\left(z\right)=exp\left\{\frac{\theta log\left(\frac{z}{\alpha }+1\right)}{1+exp\left\{-\frac{z-\mu }{\nu }\right\}}\right\},\;where\;z=\max\left(0,\;PM2.5-2.4\right)$$

where *θ*, *α*, *µ*, and *ν* are parameters that define the shape of the relationship. We selected the GEMM as it provides the most up-to-date risk estimates for the relationship between ambient air pollution and mortality globally, incorporating risk information from high-pollution regions, such as China. Additionally, the GEMM has demonstrated that mortality estimates attributable to PM exposure may be several times higher compared to those derived from other previously used risk functions, which integrate exposure data from multiple sources, including both indoor and outdoor environments [32,33]. Table S2 in Multimedia Appendix 1 provides details on the parameters of the GEMM.

### Morbidity Attributable to PM2.5 Exposure

Cardiovascular and respiratory disease–specific hospital admissions attributable to PM2.5 exposure were estimated using log-linear models. Log-linear models have previously been used in the literature for the assessment of PM2.5-related mortality and morbidity outcomes, particularly in high pollution regions, such as China [13,34,35]. Specifically, the function is defined as follows:


$$RR\left(z\right)=exp\left[\beta \left(z-z_{0}\right)\right]$$

where *β* denotes the exposure-response coefficient, representing the change in the incidence of the disease-specific outcome for each μg/m³ increase in PM2.5 concentration, and $z_{0}$ is the threshold concentration. Consistent with recent epidemiological evidence demonstrating that PM2.5 exposure can have adverse health effects even at low concentrations, no threshold concentration was applied in this analysis. Table S3 in Multimedia Appendix 1 provides an overview of the disease-specific mortality and morbidity outcomes assessed in this study, along with the corresponding exposure-response coefficients derived from national and international studies.

### Estimating Health and Economic Benefits

To estimate the economic impact of reductions in mortality and morbidity, we used the value-of-statistical life (VSL) and cost-of-illness (COI) methods. Briefly, VSL represents the monetary value assigned to reducing the risk of mortality, offering a quantitative basis for informing government decisions on environmental and health policies [36]. The estimate is derived from survey-based assessments of individuals’ stated willingness to pay for incremental reductions in mortality risk [37]. To ensure the estimates adequately reflected the risk and income specific to the Chinese context, we used VSL values derived from a recent national-level study, estimated at US $689,659 [38]. To quantify morbidity benefits, we used the COI method, which estimates the economic burden of disease [39]. Reductions in direct costs were estimated by multiplying the average hospitalization costs for cardiovascular and respiratory diseases by the projected decrease in hospital admissions under improved air quality scenarios. Baseline figures in 2020 were projected using time series regression, assuming that historical trends continued over the modeled period. We use a 5% discount rate, consistent with China’s economic evaluation guidelines [40].

In our reporting, we adhered to the GATHER (Guidelines for Accurate and Transparent Health Estimates Reporting) guidelines (Checklist 1).

### Data Source

Air quality monitoring data between January 1 and December 31, 2020, were collected from the National Environmental Monitoring Center, which contain hourly PM2.5 concentrations collected from 1497 monitoring sites in 338 Chinese cities at the prefectural level and above. In our analysis, the annual mean PM2.5 concentrations at the city level were obtained by aggregating hourly monitoring data to annual averages at each station and subsequently assigning these values to the corresponding administrative city unit by averaging across stations within each city. For baseline age-specific and cause-specific mortality in 2020, national-level estimates disaggregated by region (western, central, and eastern China) were utilized in the absence of city-level data. These estimates were derived from the Centers for Disease Control and Prevention’s mortality surveillance system, a national death registry covering over 605 counties and districts (21.1% of all counties and districts in China) [41]. Baseline mortalities under the business-as-usual scenario were projected for the modeled period using estimates from an existing study, which assessed risk factor exposures and associated noncommunicable disease mortality trends based on Global Burden of Disease data through 2030 [42]. For morbidity, baseline cardiovascular and respiratory hospitalization incidences and hospitalization costs were sourced from national and provincial statistical yearbooks and projected forward based on historical trends [43]. Moreover, age-specific and sex-specific baseline demographic data for 337 cities in 2020 were also extracted from statistical yearbooks. Table S4 in Multimedia Appendix 1 presents the primary data sources used in our health prediction model, while Table S5 in Multimedia Appendix 1 summarizes the key assumptions underlying our analyses.

### Ethical Considerations

This study is a secondary analysis of preexisting, aggregate-level data obtained from publicly available sources, including the *China Statistical Yearbook* [43] and the *China Health Statistical Yearbook* [44]. These datasets are compiled and released by the National Bureau of Statistics of China as part of routine national statistical and health surveillance. The data are reported at an aggregated city-level and do not contain individual-level data or personal identifiers. Therefore, ethics approval and informed consent were not required as it did not involve direct human participation. Permission to use the data was granted through public access, and all data were used in compliance with the terms and conditions specified by the respective data providers [44].

## Results

### PM2.5 Concentrations

**Figure 1.**
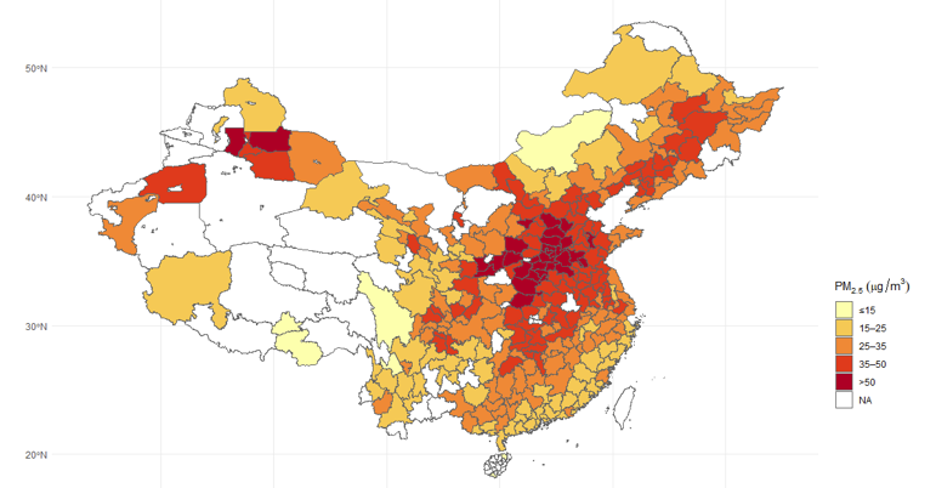
Annual average PM2.5 concentrations across Chinese prefecture-level cities in 2020. PM: particulate matter.

Figure 1 illustrates the spatial distribution of PM2.5 levels across 337 cities. In 2020, PM2.5 concentrations across 337 cities ranged from 7 to 63 µg/m³, with a national annual average of 32.6 µg/m³, falling below the 35 µg/m³ national guidelines but exceeding the WHO-recommended target of 5 µg/m³ by more than 6-fold. Higher concentrations were observed in the east compared to the west, with densely populated regions such as the Beijing-Tianjin-Hebei (BTH), Fenwei Plain (FP), and Yangtze Delta (YDR) reporting PM2.5 levels of 50.7, 47.8, and 36.7 µg/m³, respectively. Overall, our analysis accounted for nearly 70% of the total population of China in 2020.

### Mortality

**Figure 2.**
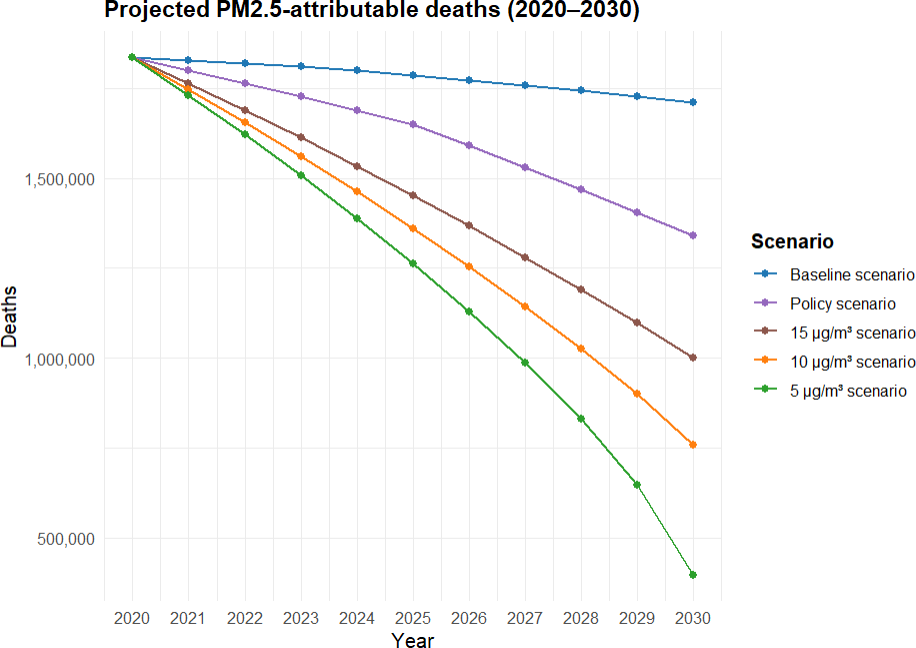
Projected PM2.5-attributable premature deaths for 337 prefecture-level cities in China (2020‐2030).

Figure 2 presents the estimated burden of premature mortality attributable to PM2.5 exposure across the modeled scenarios, based on the Shared Socioeconomic Pathway 2 (SSP2) population trajectory. Between 2021 and 2025, under the baseline scenario in which PM2.5 concentrations remained at 2020 levels, exposure was projected to contribute to 9.04 million (95% CI 7.70‐10.67 million) attributable deaths. Under the policy scenario, where concentrations were reduced by 10% from 2020 to 2025, this estimate declined to 8.63 million (95% CI 7.42‐10.30 million), corresponding to an additional 0.41 million premature deaths averted under the 14th Five-Year Clean Air Plan. Over the full modeled period from 2021 to 2030, the total number of PM2.5-attributable deaths was estimated at 17.76 million (95% CI 14.21‐20.95 million) under the baseline scenario, while the policy scenario resulted in a lower estimate of 15.96 million (95% CI 12.85‐19.15 million). If PM2.5 concentrations were further reduced to the WHO’s more stringent targets, the estimated number of attributable deaths would decline correspondingly to 13.99 million (95% CI 11.25‐15.25 million) under a 15 µg/m³ scenario, 12.86 million (95% CI 10.85‐14.85 million) under a 10 µg/m³ scenario, and 11.49 million (95% CI 8.96‐13.45 million) under a 5 µg/m³ scenario, respectively.

**Figure 3.**
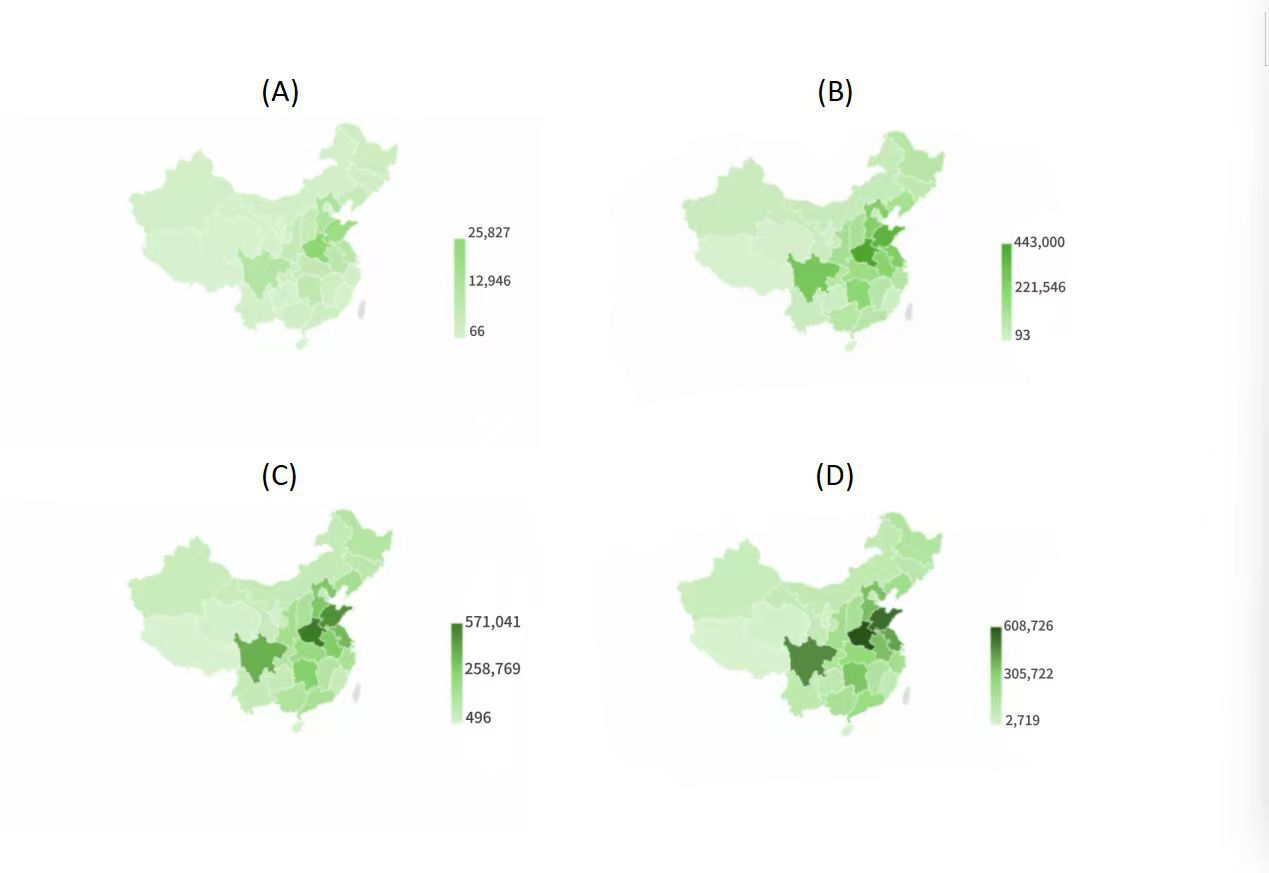
Spatial distribution of reductions in PM2.5-attributable premature deaths across Chinese prefecture-level cities under improved air quality scenarios for 2021‐2030: (A) policy scenario, (B) 15 µg/m³ target, (C) 10 µg/m³ target, and (D) 5 µg/m³ target. PM: particulate matter.

The spatial distribution of the annual average reduction in total premature deaths across provinces for each modeled scenario is shown in Figure 3. The greatest reductions in mortality were observed in the eastern and southeastern regions, particularly in highly populated and heavily polluted areas such as the BTH, FP, and YDR regions, compared to the west. Under the policy scenario, the estimated reduction in average annual premature deaths over the 10-year period was 13,879 for the BTH region, 7,009 for the FP region, and 6,895 for the YDR region. Similar spatial patterns of avoided premature deaths were observed under the WHO scenarios. In the most polluted regions (BTH, FP, and YDR), the estimated annual average benefits of reaching 15 µg/m³ corresponded to 237,580, 165,989, and 142,120 deaths averted, respectively. For the 10 µg/m³ scenario, these estimates increased to 283,878, 217,406, and 171,727, while for the 5 µg/m³ scenario, the reductions in premature deaths reached 338,916, 279,313, and 206,993, respectively.

**Figure 4.**
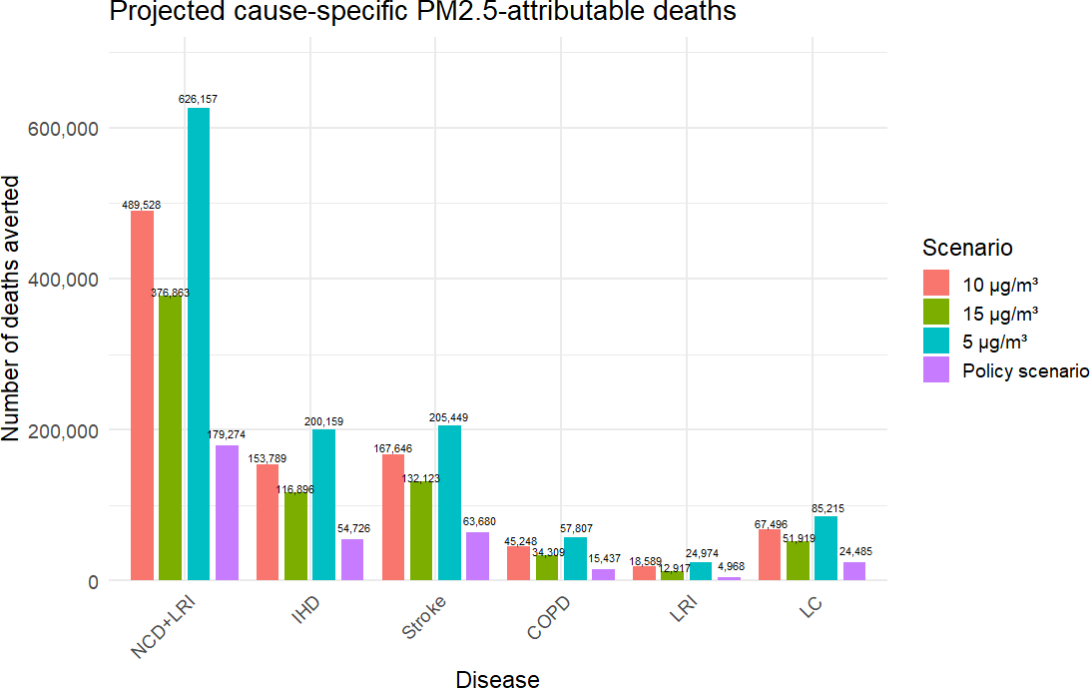
Projected annual average premature deaths by cause in China under improved air quality scenarios (2021‐2030). COPD: chronic obstructive pulmonary disease; IHD: ischemic heart disease; LC: lung cancer; LRI: lower respiratory infections; NCD: noncommunicable disease.

Figure 4 presents the annual average reduction in cause-specific premature deaths attributable to improved air quality for each modeled scenario under the SSP2 pathway. Under the policy scenario, a 10% reduction in PM2.5 concentrations by 2025, followed by a further reduction to 25 µg/m³ by 2030, is projected to result in an average annual reduction of 179,274 premature deaths. This includes 54,726 deaths from IHD, 63,680 from stroke, 15,437 from COPD, 4,968 from LRI, and 24,485 from LC. If PM2.5 concentrations were further reduced to the WHO’s interim and final targets (15.10 and 5 µg/m³), the estimated annual reductions in total premature deaths would increase to 376,863, 489,528, and 626,157, respectively. The corresponding reductions in cause-specific mortality would range from 116,896 to 200,159 for IHD, 132,123 to 205,449 for stroke, 34,309 to 57,807 for COPD, 12,917 to 24,974 for LRI, and 51,919 to 85,215 for LC.

### Morbidity

Table S6 in Multimedia Appendix 1 presents the annual average estimates of PM2.5-associated morbidity outcomes for the baseline scenario and the projected benefits under improved air quality scenarios. Under the policy scenario, the annual average morbidity benefits included 21,422 fewer cardiovascular disease admissions and 26,545 fewer respiratory disease admissions. These estimates increased to 41,690 and 51,681 cases under the 15 µg/m³ scenario, 51,884 and 64,333 cases under the 10 µg/m³ scenario, and 62,146 and 77,073 cases under the 5 µg/m³ scenario, respectively.

### Economic Benefits

**Figure 5.**
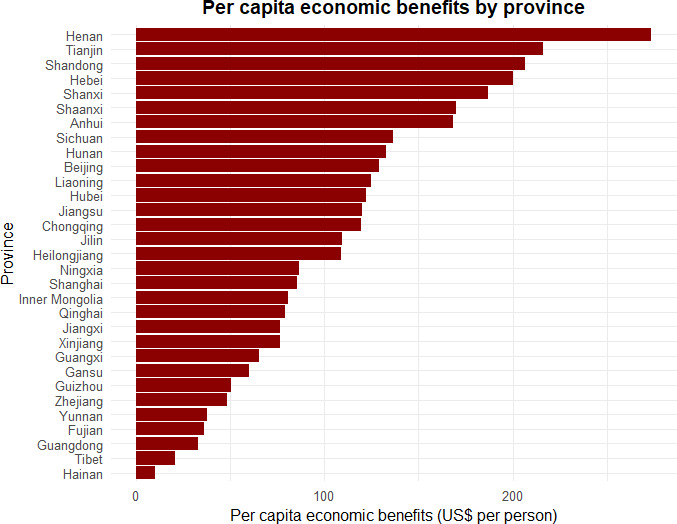
Estimated per capita economic benefits by provinces under the Healthy China 2030 Plan. USD: United States dollar.

Figure 5 illustrates the estimated per capita annual average economic benefits associated with the modeled scenarios under SSP2 at the provincial level. Under the policy scenario, the annual average economic benefits were estimated at US $123.7 billion, corresponding to approximately US $122.7 per person per year, comprising US $123.5 billion from reductions in premature mortality and US $175.5 million in annual savings from hospitalization costs. With the adoption of more stringent air quality targets of 15, 10, and 5 µg/m³, total annual savings were projected to increase to US $185.7 billion, US $240.7 billion, and US $306.5 billion, respectively. Further, the spatial distribution of economic benefits closely followed PM2.5 concentration patterns, with eastern and southeastern provinces experiencing the most substantial gains, while western regions observed relatively lower benefits. Specifically, under the policy scenario, the per capita annual average economic benefits ranged from US $10.1 in Hainan to US $273.5 in Henan, with other provinces such as Shandong (US $206.4) and Hebei (US $200.5) also realizing substantial gains. Under the more stringent targets, the average economic benefits across all 31 provinces increased progressively under similar distribution patterns, with projected benefits ranging from US $51.1 per person in Hainan to US $644.6 per person in Henan under the most stringent 5 µg/m³ target.

### Scenario Analysis

Tables S7-S10 in Multimedia Appendix 1 provide summary estimates of the mortality and morbidity outcomes under different population trajectories. Annual reductions in total premature deaths ranged from 177,857 (under SSP4) to 180,500 (under SSP3) under the policy scenario, aligning closely with the SSP2 “middle of the road” estimates. The average annual reduction in morbidity was the highest under SSP3 (48,284 cases) and lowest under SSP5 (47,588 cases). Further reductions in premature deaths were observed when achieving lower PM2.5 targets of 15, 10, and 5 µg/m³, with estimated reductions ranging from 373,985‐379,377 deaths, 485,767‐492,843 deaths, and 621,288‐630,487 deaths, respectively. Annual reductions in hospitalizations corresponded to 92,661‐93,967 cases for 15 µg/m³, 115,333‐116,965 cases for 10 µg/m³, and 138,159‐140,119 cases for 5 µg/m³. The estimated annual economic benefits under the policy scenario ranged from US $123.1 billion to US $124.9 billion, further increasing to US $184.3 to US $186.8 billion for 15 µg/m³, US $238.9 to US $242.2 billion for 10 µg/m³, and US $304.2 to US $308.5 billion for 5 µg/m³.

## Discussion

### Principal Findings

In this study, we modeled the health and economic benefits of air quality improvements in China under several population and PM2.5 concentration scenarios. Our analysis suggests that attaining China’s current national policy, which aims to reduce PM2.5 levels by 10% from 2021 to 2025 and reach 25 µg/m³ by 2030, would prevent an annual average of approximately 179,274 premature deaths and 47,967 hospitalizations, translating to economic benefits totaling US $123.7 billion (US $122.7 per capita). This would represent approximately 0.6% of China’s national GDP in 2020. Under this policy scenario, the annual average reduction in premature deaths accounted for roughly 10% of the total PM2.5-attributable deaths estimated in 2020. Reductions in deaths from IHD and stroke accounted for most of these benefits, comprising 31.4% and 34.2% of the total annual average averted deaths, respectively. Under the VSL of US $689,659, monetized mortality benefits were overwhelmingly the largest source of economic gain, comprising over 99% of the total. The greatest benefits were observed in regions with high PM2.5 concentrations and population densities, such as the BTH, FP, and YDR region. At the provincial level, Shandong, Henan, and Hebei yielded the highest estimated annual benefits, equating to 1.0%, 1.6%, and 1.4% of their respective 2020 GDPs. Moreover, our model estimates suggested that substantially greater health and economic benefits could be realized under more stringent air quality targets. Specifically, the additional annual average economic benefits were projected to be approximately 2.1, 2.7, and 3.5 times higher under the 5, 10, and 15 µg/m³ targets, respectively, compared to the national policy scenario. These results underscore the substantial potential for improved health and economic outcomes through more stringent air quality control, supporting the national government’s ongoing efforts to enhance air quality and emphasizing the necessity to set more ambitious future guidelines.

Our study contributes to the literature in several ways. First, it provides an updated analysis to support identifying at-risk populations and future policy design by projecting health and economic outcomes in the future at the national level. Limited evidence exists in this area of research. Previously, Wang et al [45] quantified the health benefits for 100% and 50% improvements in air quality based on 2010 levels, reporting a reduction in the number of PM2.5-related premature deaths in China by approximately 129,278 by 2020 and 217,988 by 2030, compared with 2010. Since then, China has significantly reduced PM2.5 concentrations, with an annual average of 32.6 µg/m³ in 2020. With the introduction of its latest 14th 5-Year Action Plan and Healthy 2030 Plan, China is set to reach the target of 25 ug/m^3^ by 2030 [22]. Additionally, in 2021, the WHO updated its air quality guidelines, recommending several updated interim and final targets to reflect the growing body of updated epidemiology evidence [19]. These updates in policies and air quality guidelines both nationally and internationally necessitate an updated analysis to support identifying at-risk populations and informing future policy design. Additionally, in this analysis, we use the GEMM to estimate mortality. Evidence shows that GEMM predicts mortality estimates over 120% higher than other established exposure-response functions, such as the WHO’s Integrated Exposure-Response model or the log-normal model by considering for epidemiological evidence specific to ambient air pollution, as well as including evidence from high pollution regions [32]. Third, our findings further contribute to the global evidence demonstrating the substantial health and economic benefits of significant reductions in ambient air pollution, particularly in highly polluted countries. For example, research from India has shown significant reductions in excess mortality for both urban and rural populations following national actions to reduce PM [12,46]. Collectively, this body of evidence encourages governments in South and South-East Asia, which already bear a disproportionate burden from high PM exposure, to establish and strengthen effective national air quality regulatory standards. Fourth, we provide an economic perspective by quantifying the potential economic benefits of air quality improvements. Using both willingness to pay and COI estimates, our analysis translates health gains into monetary terms to better inform policy and facilitate decision-making.

Several limitations and future research questions should be acknowledged. As with all modeling analyses, the accuracy of our estimates is constrained by simplifying assumptions. First, we assumed that annual average PM2.5 concentrations serve as a proxy for long-term exposure, despite individual exposure being influenced by spatial-temporal variability, socioeconomic factors, and personal health behaviors [47,48]. Second, our economic valuation heavily relied on the VSL estimate, despite the lack of a nationally recognized figure. Previous VSL studies have reported values ranging from US $23,745 to US $614,805, highlighting the significant variability [38]. In our analysis, we opted for a recent VSL figure estimated in multiple cities representative of the national population. Third, due to the absence of city-level data on age-specific population trajectories, baseline mortality, morbidity, and health care utilization, we relied on provincial and regional data from statistical yearbooks and national death registries. Fourth, future advancements in health care services could significantly alter hospitalization rates and costs, yet our projections are based on historical trends. Further, a broader societal perspective on the benefits of reducing PM2.5 could be considered. For example, this study did not incorporate indirect benefits, such as avoided productivity losses. Additionally, previous studies have indicated that air pollution mitigation can involve substantial costs, including direct abatement costs and broader social and economic impacts [30,49]. Although these were beyond the scope of this study, they may affect the overall net economic benefit of such policies and warrant further investigation. Fifth, in our analysis, although population growth was projected using the shared socioeconomic pathways, we assumed that the population age structure remained unchanged over time. This is a notable limitation in the context of China’s rapidly aging population, as epidemiological evidence indicates that older adults are more susceptible to the adverse health effects of PM [32]. Consequently, our estimates may underestimate the future disease burden attributable to air pollution and the corresponding health benefits achievable through air quality improvements. Finally, we assumed a steady decline in PM2.5 concentrations over our modeled period, while in reality, PM2.5 reductions occur through a nonlinear process.

### Conclusion

We modeled the potential health and economic benefits of improving air quality in China from 2021 to 2030. Achieving a 10% reduction in national PM2.5 concentrations by 2025, with a target of 25 µg/m³ by 2030, was estimated to prevent an average of 179,274 premature deaths and 47,967 hospital admissions annually, corresponding to US $123.7 billion in annual average economic benefits. However, more ambitious targets could yield substantially greater benefits, with annual death reductions of 376,863, 489,528, and 626,157 and hospitalization reductions of 93,371, 116,217, and 139,219, under 15, 10, and 5 µg/m³ targets, respectively, representing a 2-fold to 3-fold increase compared to the national target. Our findings underscore the substantial additional health and economic benefits of stricter air quality policies and support the development of future evidence–based national air quality guidelines.

## Supplementary material

10.2196/84809 Multimedia Appendix 1 Summary of model assumptions, parameters, and results of scenario analyses.

10.2196/84809 Checklist 1 GATHER checklist.

## Abbreviations

BTH: Beijing-Tianjin-Hebei
COI: cost-of-illness
COPD: chronic obstructive pulmonary disease
FP: Fenwei Plain
GATHER: Guidelines for Accurate and Transparent Health Estimates Reporting
GDP: gross domestic product
GEMM: global exposure mortality model
IHD: ischemic heart disease
LC: lung cancer
LRI: lower respiratory infection
NCD: noncommunicable disease
PM: particulate matter
SSP: Shared Socioeconomic Pathway
VSL: value-of-statistical life
WHO: World Health Organization
YDR: Yangtze Delta
