# Supplementary material for: Projected Health and Economic Benefits of Air Quality Targets in China: Modeling Study
Source: JMIR Public Health Surveill. 2026 Apr 1;12:e84809. doi: 10.2196/84809 (PMC13043006; doi:10.2196/84809)
Supplement: Multimedia Appendix 1 [file publichealth-v12-e84809-s001.docx]

Table 1. Summary matrix of air quality scenarios and population projections for 2021-2030

| Air quality scenarios | Population under shared socioeconomic pathways (SSPs)^[[1]](#footnote-1)^ |
| --- | --- |
| Baseline 2020 levels: assuming that annual PM2.5 concentrations remain at 2020 levels throughout the projected period. | SSP1 |
|  | SSP2 |
|  | SSP3 |
|  | SSP4 |
|  | SSP5 |
| Air action plan: a 10% reduction in PM2.5 by 2025 from 2020 levels, with stricter targets for heavily polluted regions^[[2]](#footnote-2)^, and a nationwide goal of 25 µg/m³ by 2030. | SSP1 |
|  | SSP2 |
|  | SSP3 |
|  | SSP4 |
|  | SSP5 |
| WHO levels: a reduction in PM2.5 levels to meet the WHO interim targets of 15, and 10 µg/m³ (levels III and IV), and the final target of 5 µg/m³ by 2030, respectively [1]. | SSP1 |
|  | SSP2 |
|  | SSP3 |
|  | SSP4 |
|  | SSP5 |

Table 2. Age-and-cause specific parameters for the Global Exposure Mortality Model to estimate PM2.5- attributable premature mortality in China

|  | Age range | θ | S.E. | α | µ | ν |
| --- | --- | --- | --- | --- | --- | --- |
| NCD+LRI | 27.5 | 0.1585 | 0.01477 | 1.6 | 15.5 | 36.8 |
|  | 32.5 | 0.1577 | 0.01470 | 1.6 | 15.5 | 36.8 |
|  | 37.5 | 0.1570 | 0.01463 | 1.6 | 15.5 | 36.8 |
|  | 42.5 | 0.1558 | 0.01450 | 1.6 | 15.5 | 36.8 |
|  | 47.5 | 0.1532 | 0.01425 | 1.6 | 15.5 | 36.8 |
|  | 52.5 | 0.1499 | 0.01394 | 1.6 | 15.5 | 36.8 |
|  | 57.5 | 0.1462 | 0.01361 | 1.6 | 15.5 | 36.8 |
|  | 62.5 | 0.1421 | 0.01325 | 1.6 | 15.5 | 36.8 |
|  | 67.5 | 0.1374 | 0.01284 | 1.6 | 15.5 | 36.8 |
|  | 72.5 | 0.1319 | 0.01234 | 1.6 | 15.5 | 36.8 |
|  | 77.5 | 0.1253 | 0.01174 | 1.6 | 15.5 | 36.8 |
|  | 85 | 0.1141 | 0.01071 | 1.6 | 15.5 | 36.8 |
| IHD | 27.5 | 0.5070 | 0.02458 | 1.9 | 12 | 40.2 |
|  | 32.5 | 0.4762 | 0.02309 | 1.9 | 12 | 40.2 |
|  | 37.5 | 0.4455 | 0.02160 | 1.9 | 12 | 40.2 |
|  | 42.5 | 0.4148 | 0.02011 | 1.9 | 12 | 40.2 |
|  | 47.5 | 0.3841 | 0.01862 | 1.9 | 12 | 40.2 |
|  | 52.5 | 0.3533 | 0.01713 | 1.9 | 12 | 40.2 |
|  | 57.5 | 0.3226 | 0.01564 | 1.9 | 12 | 40.2 |
|  | 62.5 | 0.2919 | 0.01415 | 1.9 | 12 | 40.2 |
|  | 67.5 | 0.2612 | 0.01266 | 1.9 | 12 | 40.2 |
|  | 72.5 | 0.2304 | 0.01117 | 1.9 | 12 | 40.2 |
|  | 77.5 | 0.1997 | 0.00968 | 1.9 | 12 | 40.2 |
|  | 85 | 0.1536 | 0.00745 | 1.9 | 12 | 40.2 |
| Stroke | 27.5 | 0.4513 | 0.11919 | 6.2 | 16.7 | 23.7 |
|  | 32.5 | 0.4240 | 0.11197 | 6.2 | 16.7 | 23.7 |
|  | 37.5 | 0.3966 | 0.10475 | 6.2 | 16.7 | 23.7 |
|  | 42.5 | 0.3693 | 0.09752 | 6.2 | 16.7 | 23.7 |
|  | 47.5 | 0.3419 | 0.09030 | 6.2 | 16.7 | 23.7 |
|  | 52.5 | 0.3146 | 0.08307 | 6.2 | 16.7 | 23.7 |
|  | 57.5 | 0.2872 | 0.07585 | 6.2 | 16.7 | 23.7 |
|  | 62.5 | 0.2598 | 0.06863 | 6.2 | 16.7 | 23.7 |
|  | 67.5 | 0.2325 | 0.06190 | 6.2 | 16.7 | 23.7 |
|  | 72.5 | 0.2051 | 0.05418 | 6.2 | 16.7 | 23.7 |
|  | 77.5 | 0.1778 | 0.04695 | 6.2 | 16.7 | 23.7 |
|  | 85 | 0.1368 | 0.03611 | 6.2 | 16.7 | 23.7 |
| COPD | >25 | 0.2510 | 0.06762 | 6.5 | 2.5 | 32 |
| Lung Cancer | >25 | 0.2942 | 0.06147 | 6.2 | 9.3 | 29.8 |
| LRI | >25 | 0.4468 | 0.11735 | 6.4 | 5.7 | 8.4 |

Table 3. Overview of disease-specific mortality and morbidity relative risk parameters used in health impact model

|  | RR per 10μg/m3 [95%CI] | Source |
| --- | --- | --- |
| Mortality |  | Burnett et al. [2] |
| NCD+LRI | GEMM (Supplementary table 3) |  |
| Stroke | GEMM (Supplementary table 3) |  |
| IHD | GEMM (Supplementary table 3) |  |
| COPD | GEMM (Supplementary table 3) |  |
| LC | GEMM (Supplementary table 3) |  |
| LRI | GEMM (Supplementary table 3) |  |
| Morbidity |  |  |
| CVD admissions | 0.037 (0.035, 0.038) | Gao et al. [3] |
| Respiratory  admissions | 0.046 (0.044, 0.048) | Gao et al. [3] |

Table 4. Identification of data sources used in the health and economic impact analysis in China

| Input | Data source | Spatial scale | Projected time period | Projection method | Purpose in the model |
| --- | --- | --- | --- | --- | --- |
| Population size and age structure | Provincial statistical yearbooks | City-level | From 2020 to 2030 | Shared socioeconomic pathways | Age-stratified health impact assessment |
| Air quality | China National Environmental Monitoring Centre | City-level | From 2020 to 2030 | 1) Policy scenario (10% reduction by 2025, 25 µg/m³ by 2030)  2) WHO interim target 1 (15 µg/m³ by 2030)  3) WHO interim target 2 (10 µg/m³ by 2030)  4) WHO guideline target (5 µg/m³ by 2030) | Exposure input for GEMM |
| Cause-specific mortality | National Disease Surveillance Points | Regional* | From 2020 to 2030 | Deaths and risk factor–attributable fractions by NCD category were projected using GBD 2013 data, with scenarios reflecting WHO risk factor reduction targets | Mortality input for GEMM |
| Casuse-specific hospitalisation incidences | Province-and-city level Health Statistical Yearbooks | Provincial level | From 2020 to 2030 | Based on historical trends | Morbidity input for log-normal model |
| Value of statistical life | Cao et al. | National | NA | NA | Quantify economic value of excess mortality |
| Hospitalisation costs | Province-and-city level Health Statistical Yearbooks | Provincial level | From 2020 to 2030 | Based on historical trends | Quantify economic value of direct medical costs |

Table 5. Identification of key model assumptions applied in the health and economic impact analysis for China

| 1. Annual average concentrations are indicative of long-term exposure |
| --- |
| 1. Air quality improves progressively throughout the modelled period. In instances where annual average concentrations are already below the target, air quality remains stable over the course of the modelled period. |
| 1. Projected cardiovascular and respiratory diseases hospital admission incidences and costs follow historical trends. |
| 1. National-and-city level age structures for remain the same during the modelled period. |
| 1. In the absence of city-specific mortality data, provincial and regional data were used. |
| 1. Population age structure remained constant over time, with age-specific population proportions fixed at 2020 baseline levels. Total population size were projected under the shared socioeconomic pathways |
| 1. Monetary value to premature deaths and hospitalisation costs were discounted at 5%. |

Table 6. Annual average estimates of PM2.5-attributable morbidity outcomes for cardiovascular disease and respiratory disease in China (2021-2030)

| Morbidity Outcome | Baseline | Policy scenario | 15 µg/m³ | 10 µg/m³ | 5 µg/m³ |
| --- | --- | --- | --- | --- | --- |
| CVD hospital admissions | 131701 | 110278 | 90011 | 79817 | 69555 |
| Respiratory hospital admissions | 163450 | 136906 | 111770 | 99118 | 86378 |
| Estimated morbidity benefits | — | 47967 | 93371 | 116217 | 139219 |

Table 7. Estimated PM2.5-attributable health and economic benefits for 337 prefecture-level cities in China under the SSP1 pathway (2021-2030)

|  | Baseline scenario | Policy scenario | 15µg/m³ target | 10µg/m³ target | 5µg/m³ target |
| --- | --- | --- | --- | --- | --- |
| NCD+LRI | 1769201 | 1590657 | 1393928 | 1281776 | 1145787 |
| IHD | 587089 | 532588 | 470690 | 433965 | 387812 |
| Stroke | 447973 | 384562 | 316416 | 281055 | 243426 |
| COPD | 154406 | 139036 | 120249 | 109365 | 96868 |
| LRI | 65334 | 60387 | 52475 | 46830 | 40476 |
| LC | 205606 | 181218 | 153900 | 138391 | 120751 |
| CVD hospitalisations | 131234 | 109903 | 89717 | 79567 | 69350 |
| Respiratory hospitalisations | 162872 | 136439 | 111404 | 98808 | 86124 |
| Estimated monetary benefits | - | $123.6 USD billion | $184.9 USD billion | $239.7 USD billion | $305.2 USD billion |

Table 8. Estimated PM2.5-attributable health and economic benefits for 337 prefecture-level cities in China under the SSP3 pathway (2021-2030)

|  | Baseline scenario | Policy scenario | 15µg/m³ target | 10µg/m³ target | 5µg/m³ target |
| --- | --- | --- | --- | --- | --- |
| NCD+LRI | 1784705 | 1604205 | 1405328 | 1291861 | 1154217 |
| IHD | 592302 | 537193 | 474607 | 437447 | 390725 |
| Stroke | 451987 | 387859 | 318963 | 283188 | 245114 |
| COPD | 155803 | 140258 | 121257 | 110237 | 97580 |
| LRI | 65910 | 60908 | 52902 | 47188 | 40755 |
| LC | 207358 | 182709 | 155100 | 139417 | 121575 |
| CVD hospitalisations | 132365 | 110800 | 90408 | 80147 | 69817 |
| Respiratory hospitalisations | 164275 | 137554 | 112263 | 99528 | 86703 |
| Estimated monetary benefits |  | $124.9 USD billion | $186.8 USD billion | $242.3 USD billion | $308.5 USD billion |

Table 9. Estimated PM2.5-attributable health and economic benefits for 337 prefecture-level cities in China under the SSP4 pathway (2021-2030)

|  | Baseline scenario | Policy scenario | 15µg/m³ target | 10µg/m³ target | 5µg/m³ target |
| --- | --- | --- | --- | --- | --- |
| NCD+LRI | 1764847 | 1586990 | 1390862 | 1279080 | 1143559 |
| IHD | 585605 | 531317 | 469613 | 433015 | 387027 |
| Stroke | 446856 | 383685 | 315748 | 280500 | 242994 |
| COPD | 154081 | 138765 | 120031 | 109177 | 96716 |
| LRI | 65179 | 60250 | 52362 | 46736 | 40402 |
| LC | 205071 | 180780 | 153549 | 138095 | 120518 |
| CVD hospitalisations | 130895 | 109642 | 89522 | 79406 | 69222 |
| Respiratory hospitalisations | 162451 | 136116 | 111163 | 98607 | 85965 |
| Estimated monetary benefits |  | $123.1 USD billion | $184.3 USD billion | $238.9 USD billion | $304.2 USD billion |

Table 10. Estimated PM2.5-attributable health and economic benefits for 337 prefecture-level cities in China under the SSP5 pathway (2021-2030)

|  | Baseline scenario | Policy scenario | 15µg/m³ target | 10µg/m³ target | 5µg/m³ target |
| --- | --- | --- | --- | --- | --- |
| NCD+LRI | 1770546 | 1591803 | 1394867 | 1282608 | 1146484 |
| IHD | 587516 | 532960 | 470999 | 434240 | 388041 |
| Stroke | 448281 | 384803 | 316598 | 281207 | 243547 |
| COPD | 154469 | 139087 | 120289 | 109399 | 96895 |
| LRI | 65383 | 60432 | 52511 | 46862 | 40502 |
| LC | 205802 | 181381 | 154028 | 138501 | 120841 |
| CVD hospitalisations | 131701 | 110001 | 89793 | 79631 | 69402 |
| Respiratory hospitalisations | 163450 | 136562 | 111499 | 98888 | 86188 |
| Estimated monetary benefits |  | $123.7 USD billion | $185.1 USD billion | $239.9 USD billion | $305.5 USD billion |

References

1. World Health Organisation. WHO global air quality guidelines: particulate matter (‎PM2.5 and PM10)‎, ozone, nitrogen dioxide, sulfur dioxide and carbon monoxide. Accessed 3 March, 2025. <https://www.who.int/publications/i/item/9789240034228>

2. Burnett R, Chen H, Szyszkowicz M, et al. Global estimates of mortality associated with long-term exposure to outdoor fine particulate matter. *Proceedings of the National Academy of Sciences*. 2018/09/18 2018;115(38):9592-9597. doi:10.1073/pnas.1803222115

3. Gao Y, Gu J, Shi Y, Wang H, Chen T, Di Q. Association of short-term exposure to ambient fine particle matter with hospital admission risks and costs in China, a case-crossover study. *Global Transitions*. 2023/01/01/ 2023;5:40-49. doi:<https://doi.org/10.1016/j.glt.2023.04.003>

1. **SSP1 (Sustainability):** A sustainable world with low inequality, strong global cooperation, a focus on renewable energy, and moderate population growth; **SSP2 (Middle of the Road)**: A world with moderate economic growth and technological progress, maintaining current trends, and moderate population growth; **SSP3 (Regional Rivalry):** A fragmented world with high regional conflicts, slow economic growth, high fossil fuel use, and steep population growth in developing countries; **SSP4 (Inequality):** A divided world with increasing inequality, and uneven population growth; **SSP5 (Fossil-fueled Development):** A world with rapid economic growth, heavy reliance on fossil fuels, fast technological advances, and high population growth. [↑](#footnote-ref-1)
2. Beijing-Tianjin-Hebei region (BTH), the Fenwei Plain (FP), and the Yangtze River Delta (YRD) [↑](#footnote-ref-2)
