# Supplementary material for: Projected Health and Economic Benefits of Air Quality Targets in China: Modeling Study
Source: JMIR Public Health Surveill. 2026 Apr 1;12:e84809. doi: 10.2196/84809 (PMC13043006; doi:10.2196/84809)
Supplement: Checklist 1 [file publichealth-v12-e84809-s002.docx]

| Item # | Checklist item | Reported on page |
| --- | --- | --- |
| Objectives and funding | | |
| 1 | Define the indicator(s), populations (including age, sex, and geographic entities), and  time period(s) for which estimates were made. | P4-6 |
| 2 | List the funding sources for the work. | P14 |
| Data Inputs | | |
| *For all data inputs from multiple sources that are synthesized as part of the study:* | | |
| 3 | Describe how the data were identified and how the data were accessed. | P4-6 |
| 4 | Specify the inclusion and exclusion criteria. Identify all ad‐hoc exclusions. | P6 |
| 5 | Provide information on all included data sources and their main characteristics. For each data source used, report reference information or contact name/institution, population represented, data collection method, year(s) of data collection, sex and age range,  diagnostic criteria or measurement method, and sample size, as relevant. | P6 and Supplementary Table 4 |
| 6 | Identify and describe any categories of input data that have potentially important biases  (e.g., based on characteristics listed in item 5). | P9 |
| *For data inputs that contribute to the analysis but were not synthesized as part of the study:* | | |
| 7 | Describe and give sources for any other data inputs. | NA |
| *For all data inputs:* | | |
| 8 | Provide all data inputs in a file format from which data can be efficiently extracted (e.g., a spreadsheet rather than a PDF), including all relevant meta‐data listed in item 5. For any data inputs that cannot be shared because of ethical or legal reasons, such as third‐party ownership, provide a contact name or the name of the institution that retains the right to  the data. | NA |
| Data analysis | | |
| 9 | Provide a conceptual overview of the data analysis method. A diagram may be helpful. | P4 |
| 10 | Provide a detailed description of all steps of the analysis, including mathematical formulae. This description should cover, as relevant, data cleaning, data pre‐processing, data adjustments and weighting of data sources, and mathematical or statistical  model(s). | P4-5 and Supplementary Tables 1-5 |
| 11 | Describe how candidate models were evaluated and how the final model(s) were  selected. | NA |
| 12 | Provide the results of an evaluation of model performance, if done, as well as the results  of any relevant sensitivity analysis. | NA |
| 13 | Describe methods for calculating uncertainty of the estimates. State which sources of  uncertainty were, and were not, accounted for in the uncertainty analysis. | Supplementary tables 1 and 3 |
| 14 | State how analytic or statistical source code used to generate estimates can be accessed. | P6 and Supplementary Table 4 |
| Results and Discussion | | |
| 15 | Provide published estimates in a file format from which data can be efficiently extracted. | Supplementary Tables 8-11 |
| 16 | Report a quantitative measure of the uncertainty of the estimates (e.g. uncertainty  intervals). | P7 |
| 17 | Interpret results in light of existing evidence. If updating a previous set of estimates,  describe the reasons for changes in estimates. | P8-9 |
| 18 | Discuss limitations of the estimates. Include a discussion of any modelling assumptions or  data limitations that affect interpretation of the estimates. | P9 |
